# Leading beyond the Script: A Cross-Sectional Study Exploring Preparedness of Pharmacy Academic Administrators

**DOI:** 10.3390/pharmacy12010025

**Published:** 2024-02-01

**Authors:** Elizabeth A. Hall, Christopher K. Finch, Katherine L. March

**Affiliations:** Department of Clinical Pharmacy and Translational Science, College of Pharmacy, The University of Tennessee Health Science Center, Memphis, TN 38163, USA; cfinch@uthsc.edu (C.K.F.); kmarch@uthsc.edu (K.L.M.)

**Keywords:** leadership, academic administration, pharmacy education, organizational efficiency, faculty, management

## Abstract

Limited research exists on the preparedness of pharmacy academic administrators for their roles. This cross-sectional survey aimed to explore the self-perceptions of pharmacy academic administrators, including deans, associate deans, assistant deans, department chairs, and directors, within United States-based Colleges or Schools of Pharmacy. Participants answered questions regarding their demographics, self-perceived readiness for administrative roles, self-perceived leadership skills, and strategies used to develop these skills. Data were analyzed using descriptive statistics, and subgroup comparisons were made using Student’s t-test for normally distributed continuous variables, Mann–Whitney tests for ordinal variables or non-normally distributed continuous variables, and Chi-squared tests for nominal variables. A total of 193 responses were analyzed. Respondents reported feeling least prepared in two areas: entrepreneurial revenue and handling grievances and appeals. There were gender differences noted in preparedness to conduct performance reviews, manage unit finances, and develop entrepreneurial revenue, with men rating themselves significantly higher than women in all three areas. Despite high self-ratings of leadership skills in the overall cohort, significant gender differences were noted in micromanagement with men rating themselves lower than women. Seeking advice from senior colleagues was the most used development strategy, and women showed a significantly higher preference for programs facilitated by professional organizations. This study contributes valuable insights into the preparedness of pharmacy academic administrators to inform future strategies that better support individuals to be successful in their roles.

## 1. Introduction

Pharmacy education relies on the leadership and expertise of academic administrators who oversee various aspects of pharmacy programs. These administrators play a vital role in shaping curricula, overseeing the recruitment and management of faculty and staff, cultivating internal and external partnerships, fostering student success, ensuring compliance with accreditation standards, engaging in budget and tuition planning, spearheading new initiatives, and collaborating with leadership at the university level, among other responsibilities [1]. The multifaceted contributions of pharmacy academic administrators are integral to the seamless functioning and advancement of pharmacy education and to ensuring the development of the next generation of pharmacists.

Individuals who excel in their faculty roles often find themselves thrust into administrative leadership positions, whether adequately prepared or not. Faculty members are focused on teaching, scholarship, and service, while administrators are typically focused more heavily on the service and administration aspect. As a result, there are very different responsibilities for faculty members versus pharmacy academic administrators, and thus faculty members may not be adequately prepared to take on this change in responsibilities. A potential side effect of inadequate preparation for the change in role is ineffective leadership.

Ineffective leadership has been noted to cause increased occupational stress and lower employee morale due to negative feelings, attitudes, and behaviors; as a result, this can decrease organizational productivity and financial gains [2,3,4,5]. Leaders must create a safe, trusting environment to establish clear boundaries, accountability structures, communication channels, appropriate hierarchy, and role clarity [6]. By proactively training pharmacy academic administrators on these leadership competencies, institutions can curb occupational stress and turnover, boost morale and financial performance, and better achieve educational outcomes.

There is a lack of research focused specifically on understanding the level of preparation for pharmacy academic administrators in their administrative roles. This study aims to address this gap by obtaining information from a national survey of pharmacy academic administrators regarding their preparation for their administrative roles. The findings of this exploratory investigation will inform strategies to better prepare and support pharmacy academic administrators to be successful in their respective roles.

## 2. Materials and Methods

### 2.1. Population and Study Design

This cross-sectional survey aimed to explore the perceptions of 1087 pharmacy academic administrators, including deans, associate deans, assistant deans, department chairs, and directors, within United States-based Colleges or Schools of Pharmacy. The target population was carefully defined to ensure the inclusion of a diverse range of administrative roles in pharmacy education. The first two questions on the instrument asked the participant to confirm that they met the inclusion criteria of currently serving as dean, associate dean, assistant dean, department chair, or director at a United States-based College or School of Pharmacy. If the participant chose “no” for either of these questions, no further questions were presented.

Email addresses of the target population were obtained from a contact list of all associate deans, assistant deans, and department chairs available from the American Association of Colleges of Pharmacy (AACP). Because the list from the AACP was lacking emails for deans at each Accreditation Council for Pharmacy Education (ACPE)-accredited institution, these were acquired from websites.

This study was reviewed by the Institutional Review Board (IRB) and determined to be eligible for exempt review under 45 CFR 46.104(d)(1). The IRB approved all research materials, including the questionnaire and consent statement, before the study commenced. Participants were fully informed about the exempt status of the study and provided voluntary informed consent by choosing to participate. No incentives were offered for participation.

### 2.2. Questionnaire Design

The questionnaire is presented in Appendix A. The items included in the questionnaire were based on findings from a previously published study of academic administrators at 145 Carnegie-ranked public research institutions [6].

Several steps were taken to ensure the validity of the questionnaire. The first step involved a thorough review of the related existing literature. This literature review aimed to identify concepts, dimensions, and relevant factors to ensure comprehensive coverage of key constructs in the instrument. Subsequently, an expert panel of pharmacy academic administrators was assembled to assess the content validity of the questionnaire. The panel was asked to assess the relevance and comprehensiveness of all items included in the questionnaire. Their feedback was instrumental in refining and revising the instrument. The questionnaire included the following two clusters: (1) preparedness for the administrative role, which was assessed by 10 items on a 5-point Likert scale, and (2) self-perceived leadership skills, which was assessed by 15 items on a 5-point Likert scale. The Likert scale ranged from “strongly disagree” (score of 1) to “strongly agree” (score of 5). The questionnaire also asked about the participants’ demographics and background, including strategies used to develop leadership and administrative skills. The final instrument validated by the panel consisted of 44 items.

The reliability of the survey instrument was assessed using Cronbach’s alpha for each of the clusters. Both clusters were noted to reliably test their respective latent constructs as the respective Cronbach’s alpha values were greater than 0.7. The Cronbach’s alpha for the first cluster (i.e., preparedness for administrative role) was 0.922 and 0.823 for the second cluster (i.e., self-ratings of leadership skills).

### 2.3. Data Collection

The questionnaire was administered to the study population online through Qualtrics (Provo, UT, USA). To prevent duplicate participation, each pharmacy academic administrator received a personalized email on 31 July 2023 with a unique link inviting them to participate in the study. Non-responders were sent two reminder emails: the first reminder email was sent 8 August 2023, and the second was sent on 16 August 2023. The collection period concluded on 31 August 2023. To uphold confidentiality, each participant’s response was treated as anonymous. The collected data were stored securely on a password-protected computer, and only authorized personnel had access to the data.

### 2.4. Data Analysis

Responses were screened for missing data, and incomplete responses were excluded from the data analysis. All statistical analyses were performed using IBM SPSS Statistics for Mac, Version 28.0 (IBM Corporation, Armonk, NY, USA). Data were summarized using descriptive statistics. Researchers pre-specified that a subgroup analysis would be conducted only for the gender variable to avoid the introduction of potential error due to multiple subgroup analyses. This particular variable was chosen in part due to the recent article by Sagraves and colleagues that noted a decline in the percentage of women fulfilling open CEO dean positions and made a call for the examination of possible barriers to entry for women [7]. Subgroup comparisons (i.e., male vs. female) were made using Student’s t-test for normally distributed continuous variables, Mann–Whitney tests for ordinal variables or non-normally distributed continuous variables, and Chi-squared tests for nominal variables. Responses from participants who chose not to disclose their gender were not included in the subgroup analysis. All statistical tests conducted were two-sided, and the alpha level used to determine statistical significance was determined to be 0.05 a priori.

## 3. Results

**Figure 1:**
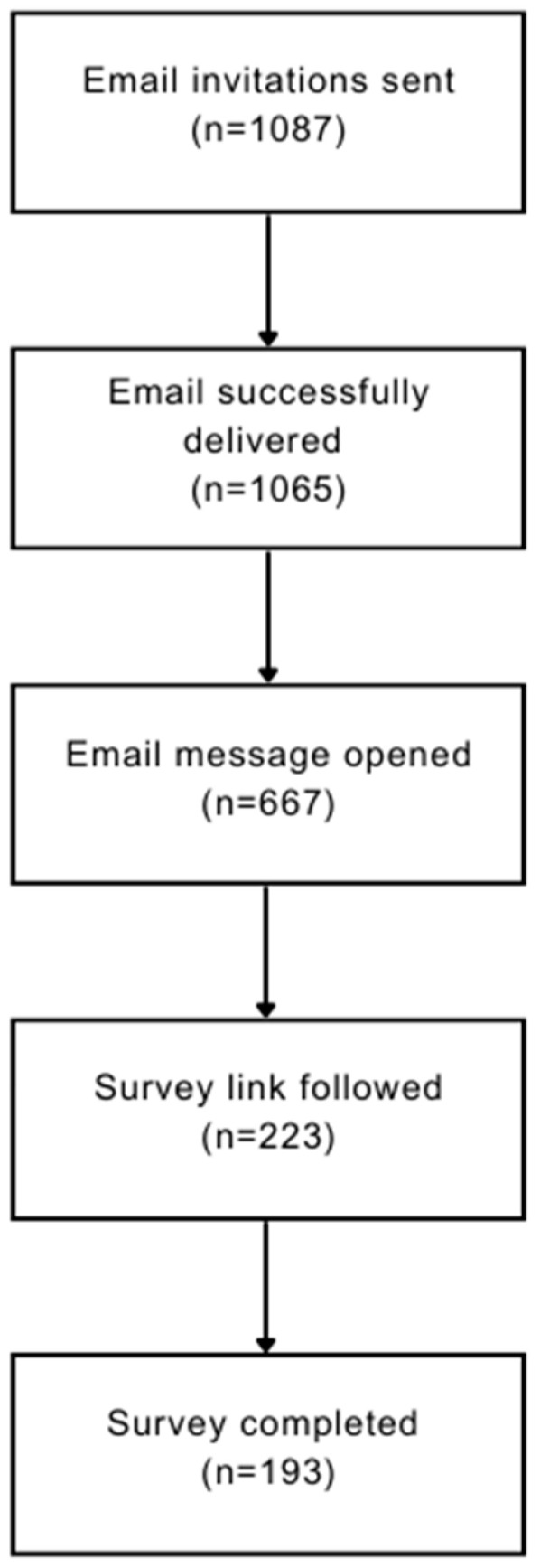
Survey response flow chart.

Email invitations to participate in the study were successfully delivered to 1065 individuals, and 193 complete responses (18.1% response rate) were included in the analysis (Figure 1). Respondent characteristics are presented in Table 1.

Perceptions of how prepared participants felt they were to begin their administrative roles are shown in Table 2. Overall, pharmacy academic administrators felt they had been least well prepared in the areas of entrepreneurial revenue and handling grievances and appeals. Notably, preparedness to conduct performance reviews saw a significant gender-related discrepancy, with men rating themselves significantly higher than women. Similarly, managing unit finances and entrepreneurial revenue showed significant gender variations, with men rating themselves higher in both cases.

Participants’ self-perceived ratings of leadership skills are shown in Table 3. No statistically significant differences were observed between men and women for 14 of the 15 individual characteristics. The one characteristic showing a gender difference was micromanagement, and men scored themselves significantly lower than women.

Strategies used to improve or develop their leadership and administrative skills are displayed in Table 4. Seeking advice from senior colleagues emerged as the most commonly utilized approach across the overall cohort. Seminars and programs facilitated by professional organizations were significantly more popular among women compared to men. Significantly more women than men reported using “other” strategies to develop their skills. Other strategies included seeking advice from peer colleagues (n = 7), graduate coursework (n = 4), local leadership fellows program (n = 3), previous administrative experience (n = 3), certificate programs (n = 2), podcasts (n = 2), and mentoring others (n = 2).

## 4. Discussion

The findings of this cross-sectional survey provide valuable insights into the preparedness of pharmacy academic administrators to fulfill their roles effectively. This study specifically explored the self-perceptions of preparedness of pharmacy academic administrators within United States-based Colleges or Schools of Pharmacy, as well as strategies employed to develop these skills. By conducting subgroup analyses stratified by gender, this research also contributes nuanced insights into the distinctive experiences encountered by male and female pharmacy academic administrators to further enrich our understanding of the multifaceted dynamics and challenges faced by these individuals.

### 4.1. Self-Ratings of Preparedness for Administrative Role

In this study, participants rated themselves relatively low in preparedness to handle grievances and appeals, emphasizing a notable deficiency in navigating conflict resolution processes. This inadequacy may be attributed to the common trajectory of individuals transitioning from faculty roles to administrative positions. The current academic structure often falls short of providing adequate opportunities for faculty members to acquire essential skills for effective conflict resolution. Furthermore, there are very few training programs in academic pharmacy that aim to prepare the next generation of administrators. Given that approximately 20% of chairs and deans are vacating their roles annually in higher education, the existing leadership development programs prove insufficient [6]. This underscores the urgent need for the development of additional strategic interventions, particularly targeted training programs. Addressing this gap through focused training initiatives could contribute substantially to the professional development of academic administrators and better prepare them for the challenges inherent in their roles, including handling grievances and appeals.

A recent publication describes the use of one such innovative and strategic intervention: article clubs [8]. During these article clubs, leadership concepts described in Harvard Business Review’s top 10 leadership articles [9] were discussed in an open forum for all faculty. This tactic proved beneficial in promoting collegiality and increasing awareness of novel leadership concepts among junior faculty. An approach similar to this one could provide a valuable avenue for administrators to engage in targeted discussions to enhance the development of specific leadership skills among current faculty members to ensure preparedness for administrative roles.

Women may have reported lower levels of preparedness in conducting performance reviews for several interconnected reasons. One significant factor is the confidence gap that persists between men and women, where societal expectations and stereotypes may contribute to women feeling less assured in leadership roles [10]. Additionally, women in executive leadership roles are more prone to experiencing imposter syndrome, a phenomenon where individuals doubt their accomplishments and fear being exposed as frauds, which may lead to a diminished sense of readiness for tasks such as conducting performance evaluations [11]. Societal gender stereotypes also likely play a crucial role, reinforcing traditional expectations that women are nurturing and less assertive, which can impact perceptions of their preparedness for authoritative tasks [12]. Addressing these challenges requires dismantling stereotypes, promoting confidence-building initiatives, and fostering inclusive environments that value diverse leadership styles.

The lower reported level of preparedness among female participants in managing unit finances and generating entrepreneurial revenue is a trend that aligns with existing research highlighting gender-based differences in financial literacy levels, as evidenced in previous studies [13,14]. Recognizing this disparity is essential in addressing the broader issue of gender equity in leadership within pharmacy education. The findings herein accentuate the importance of enhancing financial literacy skills among female administrators to mitigate the observed gap. By fostering a more inclusive and equitable approach to financial management education, academic institutions can proactively contribute to leveling the playing field and promoting a more balanced representation of pharmacy academic administrators.

### 4.2. Self-Perceptions of Leadership Skills

Overall, study participants, irrespective of gender, exhibited high levels of self-perceived leadership skills. This positive self-assessment is likely rooted in the multifaceted nature of responsibilities inherent to pharmacy academic administration, requiring a blend of organizational acumen, strategic vision, and interpersonal skills [1]. It signifies an inherent understanding, both individually and collectively, of the leadership competencies required to navigate the intricate landscape of higher education institutions.

While the positive self-assessments suggest pharmacy education administrators perceive themselves as competent leaders, these self-perceptions must be interpreted with caution. Administrators may rate themselves higher on technical expertise than soft skills like relationship building [15]. A competency gap analysis incorporating multi-rater feedback would provide a more accurate picture of development needs and help administrators calibrate their self-assessments of their leadership and administrative skills [16]. Training programs addressing hubristic biases may also help leaders develop greater self-awareness and avoid the pitfalls of overconfidence.

The observed gender differences in specific leadership skill areas emphasize the need for professional development opportunities that address the unique challenges faced by both male and female administrators. While the study did not identify significant gender differences in most leadership skills, a notable exception was observed around micromanagement, where men scored themselves significantly lower than women. Having a better understanding of the factors contributing to these gender-specific perceptions of micromanagement could provide insights into the leadership dynamics within pharmacy education. A scoping review examining micromanagement in clinical supervision across health professions education evaluated factors contributing to micromanagement [17]. The review identified contributing factors to be distrust, perfectionism, and low self-esteem. This may offer intriguing comparisons when exploring the gender-specific perceptions of micromanagement among pharmacy academic administrators in the current study. The identified solutions in the clinical supervision context were entrusting autonomy, clear communication, and organizational management [17]. It is important to recognize micromanagement as a phenomenon with consequences reaching beyond individual well-being, including organizational dysfunction and poorer employee performance [18].

### 4.3. Strategies Used for Skill Development

Seeking advice from senior colleagues emerged as the most utilized approach to enhance leadership and administrative skills, accentuating the importance of mentorship and knowledge sharing within the pharmacy academic community that has been previously published [19,20,21,22,23]. A strategy to consider for leadership development that is closely related to mentorship is intentional succession planning. A recent article noted that all administrators in pharmacy education have a critical role in developing talent and establishing robust succession plans [24]. Succession planning goes beyond replacing individuals and focusing on the highest-ranked leadership positions; organizations should instead broaden their perspectives to all levels within the organization and explore the potential in all faculty to avoid costly leadership gaps and losses.

The current study noted gender differences in the preference for seminars and programs facilitated by professional organizations. Specifically, women showed a significantly higher inclination towards such programs compared to men. This suggests that individualized development opportunities that acknowledge and address gender-specific needs would be beneficial in fostering an inclusive leadership environment within pharmacy education. This aligns with recent evaluations of gender equity in pharmacy education that have identified the importance of mentorship and support for women throughout their careers, especially in areas such as academic advancement, grant applications, salary negotiation, leadership pursuit, and award applications [25]. The observed gender variations in program preference indicate a possible need for novel initiatives that recognize and cater to diverse developmental needs within the academic administrative community, thereby contributing to a more equitable and supportive landscape for pharmacy leaders. Leadership development opportunities must extend beyond general “skills-building” to conceptualize leadership within a gendered social context [26].

Nearly one-third of the study participants reported that they had participated in the Academic Leadership Fellows Program (ALFP) offered by the AACP to develop their skills [27]. This year-long program aims to cultivate the next generation of leaders in pharmacy education. Annually selecting up to 35 fellows through a competitive application process, the ALFP delivers targeted leadership development encompassing self-assessment, mentorship networks, team exercises, and exposure to real-world administrative challenges. The curriculum covers critical competencies like strategic planning, change management, budgeting, and interpersonal skills. While not a substitute for years of progressive experience, the ALFP accelerates leadership readiness by directly building the core aptitudes needed for effective pharmacy academic administration. With its strong track record and curriculum aligned to leadership objectives, the ALFP makes an invaluable contribution to expanding leadership capacity. However, the program is limited to one individual per institution and thus may not be the panacea to address the number of administrative leaders needed in pharmacy education.

### 4.4. Limitations

While this study provides valuable insights, certain limitations should be considered. The self-reported nature of the data may introduce bias, and the cross-sectional design limits the establishment of causality. Another notable limitation is the incomplete gender data for a subset of respondents as 13.5% of the study population chose not to specify their gender. As a result, the impact of missing gender data cannot be entirely disregarded and should be considered when interpreting the subgroup comparisons.

The study’s response rate of 18.1% poses a potential limitation as it may introduce a selection bias, whereby the perspectives of non-responders are not captured. This could also impact the generalizability of the findings to the broader population of pharmacy academic administrators. However, the study sample herein is generally reflective of the demographics of the overall population, as noted by the data available in the 2022–2023 AACP Profile of Pharmacy Faculty [28]. The study population predominantly aligns with the age distribution observed in pharmacy academic administrators across the United States, with the largest contingent falling within the 50–59 years age bracket, mirroring the mean age of 52.3 years in the study sample. Similarly, the gender distribution in the study sample closely resembles national trends, with approximately 53% of all pharmacy academic administrators in the US being female (48% in the study sample). Further breakdowns indicate consistent representation, with deans at 34% female (36% in the study sample), associate deans at 54% female (59% in the study sample), and assistant deans at 61% female (67% in the study sample). Moreover, the study sample revealed that a substantial proportion held tenured positions (41%), in line with the national population. The distribution of the highest degree earned, with 54.4% holding a PharmD, is also reflective of the general population (50.8% with PharmD as the terminal degree). Of note, the AACP does not provide demographic data for individuals serving as department chairs or directors, and thus comparisons for these specific subgroups cannot be made [28].

### 4.5. Future Directions

Future research endeavors should adopt longitudinal approaches to track the development of leadership skills over an extended period. By adopting such longitudinal frameworks, researchers can not only capture the immediate impact of leadership development but also discern nuanced changes and patterns that unfold over time. This longitudinal perspective would offer valuable insights into the sustained growth and adaptation of leadership competencies among pharmacy academic administrators.

In addition to longitudinal approaches, qualitative investigations present an opportunity to delve deeper into the factors influencing perceived preparedness and leadership dynamics. Qualitative research methods, such as interviews or focus groups, can capture the rich, context-specific nuances that quantitative surveys may not fully capture. These methods could provide a deeper understanding of the lived experiences of pharmacy academic administrators, shedding light on the contextual factors that shape their leadership journeys. This approach has the potential to illuminate the contextual factors that intricately shape leadership journeys, offering a more comprehensive portrayal of the challenges, triumphs, and unique circumstances that influence the development of pharmacy academic administrators.

## 5. Conclusions

This study contributes to the limited literature on pharmacy academic administrators’ preparedness for and experience in their roles. By addressing the existing gap in the literature, this study not only sheds light on the challenges faced by these administrators but also underscores the importance of recognizing gender differences in their preparedness levels. The identification of specific areas, such as entrepreneurial revenue and conflict resolution, where administrators feel less prepared offers a strategic entry point for targeted interventions. Academic institutions and professional organizations can leverage these findings to develop and refine leadership development programs that specifically address the nuanced needs of pharmacy academic administrators.

## Figures and Tables

**Table 1:** Respondent characteristics.

Characteristic ^1^	Total (n = 193)	Men (n = 74)	Women (n = 93)
Age in years, mean (SD)	52.3 (8.52)	54.1 (8.07)	50.2 (7.85)
Gender, n (%)		n/a	n/a
Male	74 (38.3%)		
Female	93 (48.2%)		
Not specified	26 (13.5%)		
Current Administrative Role, n (%)			
Dean	25 (13.0%)	15 (20.3%)	9 (9.7%)
Associate Dean	70 (36.3%)	28 (37.8%)	41 (44.1%)
Assistant Dean	30 (15.5%)	10 (13.5%)	20 (21.5%)
Department Chair	42 (21.8%)	18 (24.3%)	22 (23.7%)
Director	4 (2.1%)	3 (4.1%)	1 (1.1%)
Tenure Status, n (%)			
Tenured	79 (40.9%)	41 (55.4%)	37 (39.8%)
Tenure track	4 (2.1%)	3 (4.1%)	1 (1.1%)
Nontenure track	62 (32.1%)	18 (24.3%)	43 (46.2%)
Nontenure institution	24 (12.4%)	11 (14.9%)	11 (11.8%)
Faculty Rank Upon Entry into Administration, n (%)			
Instructor	5 (2.6%)	2 (2.7%)	3 (3.2%)
Assistant Professor	29 (15.0%)	12 (16.2%)	16 (17.2%)
Associate Professor	79 (40.9%)	30 (40.5%)	49 (52.7%)
Professor	52 (26.9%)	28 (37.8%)	21 (22.6%)
Previous Administrative Role Outside of Academia, n (%)	51 (26.4%)	23 (31.1%)	25 (26.9%)
Number of Previous Administrative Roles, median (range)	2 (0–15)	1.5 (0–15)	2 (0–10)
Years in Current Position, median (range)	5 (0–39)	6 (1–39)	4 (1–25)
Years in Administration, median (range)	11 (1–45)	13 (1–45)	10 (1–40)
Number of Institutions Served in Administrative Roles, median (range)	1 (1–7)	2 (1–7)	1 (1–7)
Currently in Interim Role, n (%)	7 (3.6%)	2 (2.7%)	4 (4.3%)
Highest Degree Earned, n (%)			
Baccalaureate	2 (1.0%)	0 (0%)	2 (2.2%)
Masters	2 (1.0%)	1 (1.4%)	1 (1.1%)
PharmD	105 (54.4%)	40 (54.1%)	63 (67.7%)
PhD	54 (28.0%)	31 (41.9%)	21 (22.6%)
Other doctorate	8 (4.1%)	2 (2.7%)	6 (6.5%)
Post-graduate Training Completed, n (%) ^2^			
PGY1 ^3^	66 (34.2%)	21 (28.4%)	45 (48.4%)
PGY2 ^3^	36 (18.7%)	14 (18.9%)	21 (22.6%)
Academia Fellowship	10 (5.2%)	7 (9.5%)	3 (3.2%)
Research Fellowship	40 (20.7%)	25 (33.8%)	13 (14.0%)
Other	19 (9.8%)	8 (10.8%)	9 (9.7%)
Completed coursework in, n (%) ^2^			
Business administration	40 (20.7%)	14 (18.9%)	25 (26.9%)
Human resources/leadership	35 (18.1%)	13 (17.6%)	21 (22.6%)
Industrial-organizational psychology	13 (6.7%)	6 (8.1%)	6 (6.5%)
Behavioral psychology	19 (9.8%)	6 (8.1%)	12 (12.9%)
None of these	110 (57.0%)	51 (68.9%)	56 (60.2%)

^1^ Not all respondents provided demographic information and thus category percentages may not equal 100%. ^2^ Respondents could select more than one option. ^3^ Of those who chose PGY1 and/or PGY2, one indicated it to be an administration-focused residency.

**Table 2:** Self-ratings of preparedness for administrative role.

Characteristic, Mean (SD)	Total (n = 193)	Men (n = 74)	Women (n = 93)	*p*-Value
Managing staff members	3.16 (1.22)	3.34 (1.13)	2.98 (1.28)	NS
Conducting performance reviews	3.15 (1.21)	3.36 (1.19)	2.92 (1.22)	0.020
Handling grievances and appeals	2.73 (1.22)	2.88 (1.30)	2.57 (1.16)	NS
Running efficient meetings	3.79 (1.07)	3.73 (1.08)	3.78 (1.07)	NS
Allocating limited resources	3.38 (1.14)	3.49 (1.13)	3.18 (1.18)	NS
Managing unit’s finances	3.01 (1.35)	3.24 (1.20)	2.64 (1.39)	0.004
Developing entrepreneurial revenue	2.41 (1.33)	2.62 (1.34)	2.02 (1.19)	0.003
Balancing requests	3.37 (1.02)	3.46 (0.98)	3.21 (1.08)	NS
Setting strategic goals	3.54 (1.08)	3.62 (0.96)	3.37 (1.18)	NS
Developing metrics	3.39 (1.16)	3.43 (1.14)	3.23 (1.21)	NS

**Table 3:** Self-ratings of leadership skills.

Characteristic, Mean (SD)	Total (n = 193)	Men (n = 74)	Women (n = 93)	*p*-Value
Sets clear expectations	4.19 (0.65)	4.22 (0.60)	4.14 (0.72)	NS
Matches actions and words	4.62 (0.55)	4.68 (0.55)	4.57 (0.55)	NS
Follows through on commitments	4.69 (0.52)	4.65 (0.56)	4.74 (0.46)	NS
Is proactive	4.33 (0.73)	4.19 (0.81)	4.40 (0.68)	NS
Focuses on critical activities	4.34 (0.70)	4.39 (0.59)	4.29 (0.70)	NS
Assesses poor performance objectively	3.97 (0.81)	4.07 (0.76)	3.88 (0.84)	NS
Is someone others want to follow	4.09 (0.78)	4.05 (0.78)	4.10 (0.79)	NS
Provides helpful feedback	4.24 (0.59)	4.22 (0.63)	4.27 (0.55)	NS
Comfortable leading change	4.30 (0.79)	4.34 (0.76)	4.25 (0.84)	NS
Open to feedback	4.52 (0.56)	4.54 (0.53)	4.51 (0.60)	NS
Uses meeting time effectively	4.13 (0.84)	4.19 (0.81)	4.10 (0.83)	NS
Inspires others	3.88 (0.80)	3.90 (0.77)	3.87 (0.84)	NS
Micromanages the work of others	1.60 (0.71)	1.47 (0.74)	1.73 (0.68)	0.004
Talks rather than listens	2.24 (0.97)	2.31 (0.99)	2.19 (0.92)	NS
Avoids making decisions	1.75 (0.81)	1.81 (0.92)	1.74 (0.71)	NS

**Table 4:** Strategies used to develop leadership and administrative skills.

Characteristic, Mean (SD)	Total (n = 193)	Men (n = 74)	Women (n = 93)	*p*-Value
Seeking advice from senior colleagues	159 (82.4%)	70 (94.6%)	89 (95.7%)	NS
Reading about administration and leadership	138 (71.5%)	60 (81.1%)	78 (83.9%)	NS
Institutionally mandated seminars or workshops	77 (39.9%)	33 (44.6%)	44 (47.3%)	NS
Seminars and programs through professional organizations	136 (70.5%)	53 (71.6%)	83 (89.2%)	0.005
Optional seminars through institution	80 (41.5%)	29 (39.2%)	51 (54.8%)	NS
Paid professional consultation services	15 (7.8%)	6 (8.1%)	9 (9.7%)	NS
Career coaching services	18 (9.3%)	7 (9.5%)	11 (11.8%)	NS
AACP Academic Leadership Fellows Program	60 (31.1%)	26 (35.1%)	34 (36.6%)	NS
Other	23 (11.9%)	3 (4.1%)	20 (21.5%)	0.001

## Data Availability

Data are not available for sharing due to ethical, legal, and privacy restrictions.

## Appendix A. Questionnaire Assessing Preparedness of Pharmacy Academic Administrators

Q1 I am an administrator at a College or School of Pharmacy located in the United States.

O Yes (1)

O No (2)

Q2 I am currently serving as a dean, associate dean, assistant dean, department chair, or director at my College or School of Pharmacy.

O Yes (1)

O No (2)

Q3 Please indicate to what extent you felt prepared for each of the following areas of responsibility prior to beginning your current administrative position.

	**Not well at all (1)**	**Slightly well (2)**	**Moderately well (3)**	**Very well (4)**	**Extremely well (5)**
▪ Managing staff members	○	○	○	○	○
▪ Performance reviews	○	○	○	○	○
▪ Grievances and appeals	○	○	○	○	○
▪ Running efficient meetings	○	○	○	○	○
▪ Allocating limited resources	○	○	○	○	○
▪ Managing unit’s finances	○	○	○	○	○
▪ Entrepreneurial revenue	○	○	○	○	○
▪ Balancing requests	○	○	○	○	○
▪ Setting strategic goals	○	○	○	○	○
▪ Developing metrics	○	○	○	○	○

Q4 Please indicate the degree of your agreement with each statement below.

	**Strongly disagree (1)**	**Somewhat disagree (2)**	**Neither agree nor disagree (3)**	**Somewhat agree (4)**	**Strongly agree (5)**
▪ I set clear expectations.	○	○	○	○	○
▪ I match my actions and words.	○	○	○	○	○
▪ I follow through on commitments.	○	○	○	○	○
▪ I am proactive.	○	○	○	○	○
▪ I focus on critical activities.	○	○	○	○	○
▪ I address poor performance objectively.	○	○	○	○	○
▪ I am someone others want to follow.	○	○	○	○	○
▪ I provide helpful feedback.	○	○	○	○	○
▪ I am comfortable leading change.	○	○	○	○	○
▪ I am open to feedback.	○	○	○	○	○
▪ I use meeting time effectively.	○	○	○	○	○
▪ I inspire others.	○	○	○	○	○
▪ I micromanage the work of others.	○	○	○	○	○
▪ I talk rather than listen.	○	○	○	○	○
▪ I avoid making decisions.	○	○	○	○	○

Q5 From the list below, select all of the strategies you have used to develop or improve your leadership and administrative skills.

□ Seeking advice from senior colleagues (1)
□ Reading about administration and leadership (2)
□ Institutionally mandated seminars/workshops (3)
□ Seminars and programs through professional organizations (4)
Optional seminars through institution (5)
□ Paid professional consultation services (6)
□ Career coaching services (7)
□ AACP Academic Leadership Fellows Program (ALFP) (8)
□ Other (9) __________________________________________________
□ None (10)

Q6 Gender

o Male (1)
o Female (2)
o Non-binary (3)
o Prefer not to say (4)

Q7 Age: _______

Q8 Current Administrative Role

o Dean (1)
o Associate Dean (2)
o Assistant Dean (3)
o Department Chair (4)
o Director (5)

Q9 Highest degree earned

o Baccalaureate (BS, BA, etc.) (1)
o Masters (MS, MA, etc.) (2)
o PharmD (3)
o PhD (4)
o Other doctorate (5)

Q10 Post-graduate training completed

□ Post-graduate year one residency (1)
□ Post-graduate year two residency (2)
□ Fellowship focused in academia (3)
□ Fellowship focused in research (4)
□ Other (5) __________________________________________________

*Display Q11:*

*If Post-graduate training completed = Post-graduate year one residency*

*Or Post-graduate training completed = Post-graduate year two residency*

Q12 Was your post-graduate residency training administration-focused?

o Yes (1)
o No (2)

Q13 Tenure status

o Tenured (1)
o Tenure track (2)
o Nontenure track (3)
o Nontenure institution (4)

Q14 Have you taken undergraduate or graduate coursework in any of the following?

□ Business administration (1)
□ Human resources/leadership (2)
□ Industrial-organizational psychology (3)
□ Behavioral psychology (4)
□ None (5)

Q15 What was your faculty rank when you started in an administrative role?

o Instructor (1)
o Assistant Professor (2)
o Associate Professor (3)
o Professor (4)

Q16 Have you previously worked in an administrative role outside of academia?

o Yes (1)
o No (2)

Q17 How many previous administrative roles have you held?

________________________________________________________________

Q18 How many years have you been in your current position?

________________________________________________________________

Q19 How many years have you been in administration (all positions)?

________________________________________________________________

Q20 For the administrative portion of your career, how many different institutions have you served?

________________________________________________________________

Q21 Is your current administrative role an interim position?

o Yes (1)
o No (2)
